# Neuropsychological Assessment in Patients with Traumatic Brain Injury: A Comprehensive Review with Clinical Recommendations

**DOI:** 10.3390/biomedicines11071991

**Published:** 2023-07-14

**Authors:** William Torregrossa, Michele Torrisi, Rosaria De Luca, Carmela Casella, Carmela Rifici, Mirjam Bonanno, Rocco Salvatore Calabrò

**Affiliations:** 1 IRCCS Centro Neurolesi “Bonino Pulejo”, Via Palermo Cda Casazza, SS113, 98124 Messina, Italy; william.torregrossa@irccsme.it (W.T.); michele.torrisi@irccsme.it (M.T.); rosaria.deluca@irccsme.it (R.D.L.); carmela.rifici@irccsme.it (C.R.); roccos.calabro@irccsme.it (R.S.C.); 2 Department of Clinical and Experimental Medicine “AOU Policlinico G. Martino”, University Hospital “G. Martino”, 98124 Messina, Italy; ccasella@unime.it

**Keywords:** neuropsychology, traumatic brain injury, disorder of consciousness, cognitive assessment

## Abstract

Traumatic brain injury is damage to the brain occurring after birth, often resulting in the deterioration of cognitive, behavioural, and emotional functions. Neuropsychological evaluation can assist clinicians to better assess the patient’s clinical condition, reach differential diagnoses, and develop interventional strategies. However, considering the multiple rating scales available, it is not easy to establish which tool is most suitable for the different brain injury conditions. The aim of this review is to investigate and describe the most used neurocognitive assessment tools in patients with traumatic brain injury to provide clinicians with clear indications on their use in clinical practice. Indeed, during the acute phase, after the head trauma, alertness and wakefulness of the patients affected by a disorder of consciousness can be assessed using different scales, such as the Coma Recovery Scale-Revised. In both postacute and chronic phases after traumatic brain injury, general cognitive assessment tools (such as the Mini Mental State Examination) or more specific cognitive tests (e.g., Wisconsin Card Sorting Test and Trail Making Test) could be administered according to the patient’s functional status. In this way, clinicians may be aware of the patient’s neuropsychological and cognitive level, so they can guarantee a personalized and tailored rehabilitation approach in this frail patient population.

## 1. Introduction

Traumatic brain injury (TBI) is an acquired insult to the brain from an external mechanical force that may result in a temporary or permanent impairment of motor and cognitive functions [1]. It represents the leading cause of mortality and disability among young individuals in western countries [2]. TBI is classified as mild, moderate, or severe depending on the level of consciousness and the duration of coma and post-traumatic amnesia (PTA) [3]. TBI is considered as severe (sTBI) when it causes a condition of coma protracted over time (GCS ≤ 8 lasting more than 24 h). Following sTBI, patients often show long-term alterations in their state of consciousness. Briefly, the state of coma is the condition that occurs after a head injury or from the temporary absence of oxygen to the brain, where the patient lacks any residual consciousness. From this state, it is possible to achieve a nearly complete recovery or to evolve into a disorder of consciousness (DOC), like a vegetative state (more recently named unresponsive wakefulness syndrome, UWS), in which the patient regains a sleep–wake cycle although he or she is not aware of him/herself or of the surrounding environment [4]. Sometimes, such patients can partially reacquire a state of awareness and this state is called a Minimally Conscious State (MCS).

The awakening process, with an improvement in awareness and consciousness, is related to the location and extent of the brain lesion [5]. Notably, within the UWS group, the traumatic causes have a better prognosis than nontraumatic ones, given that recent studies show long-term recovery over a year after injury [6]. On the contrary, cognitive recovery tends to be rapid in patients with mild to moderate TBI [7,8]. Even after the recovery of consciousness, the main dysfunctions following TBI are (i) sensory–motor impairments, (ii) cognitive deficits involving attentional processes, executive functions, memory abilities, reasoning and problem-solving, and linguistic and visual–spatial cognition; and (iii) behavioural alterations, such as apathy, irritability, aggression, disinhibition, inertia, and mood disorders [9,10]. These symptoms are also frequently associated with anosognosia [11] and alexithymia, negatively affecting emotional status and awareness of the condition [12].

A careful and constant clinical observation, including a cognitive assessment, is critical in guiding clinicians in the assessment of the level of consciousness (UWS vs. MCS), as well as in determining the prognosis and the likelihood to recover cognitive abilities [13]. In particular, in the acute phase of sTBI, only observational scales, such as the Glasgow coma scale (GCS), the Disability Rating Scale (DRS), the Coma Recovery Scale-Revised (CRS-R), and Level of Cognitive Functioning (LCF), can be administered, since the patient can only be subjected to very simple requests or observed in spontaneous behaviours. Further neurophysiological investigation of the specific cognitive domains may be carried out only if the patient passes the state of MCS, as it needs the active participation of the subject [14]. Neuropsychological assessment in TBI patients requires a comprehensive approach to investigate the main cognitive abilities, psychiatric symptoms [15], as well as psychological factors [16]. This issue has already been investigated in previous reviews. In 2010, Podell et al. [17] described both cognitive and psychiatric symptoms of TBI (mild, moderate, and severe) globally, reporting also the neuropsychological and psychiatric evaluation tools. In a similar way, Soble et al. [18] reported the neuropsychological assessment routinely used in clinical settings in TBI patients, but without specifying the severity level of brain injury. Despite the variety in the literature in this field, research into sTBI and its neuropsychological assessment is still poor. In fact, our aim was to investigate and update the most used neuropsychological tools for cognitive assessment in patients with moderate-to-severe TBI, including those with DOC, to provide clinicians with indications on their use in current clinical practice.

In detail, the major contributions of our research consists of

- Individuating the most-used assessment tools for neuropsychological impairments after severe TBI as well as the screening tools for early cognitive evaluation during the acute phase, when the level of consciousness is altered.
- Highlighting the importance of assessment in clinical practice to achieve the most personalized and tailored rehabilitation intervention, both conventional and/or advanced.
- Providing a rationale for clinical advice about the choice of neuropsychological tools and the makeup of the clinical setting, for those who are new to this topic, based on the latest literature.

## 2. Materials and Methods

### Search Strategy and Study Selection

**Figure 1:**
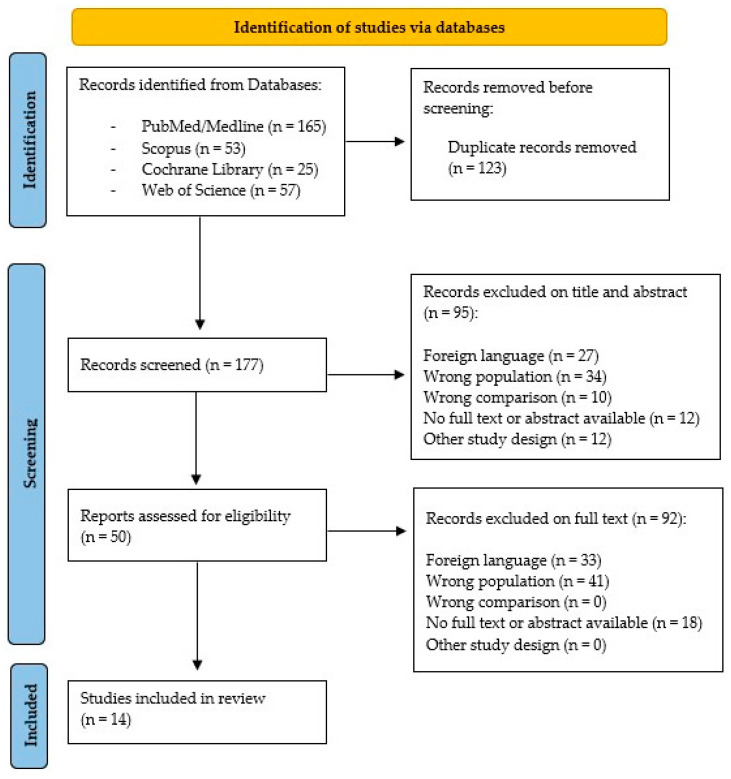
PRISMA flow-chart for the study selection.

Our scoping review followed the acronym of PCC (population/problem, concept, context). We considered adult patients affected by moderate and severe TBI as the population/problem, the concept was the use of neuropsychological assessment tools, while the context was the neurorehabilitation setting. The studies included in our review were obtained through a search on PubMed/Medline, Scopus, Cochrane Library, and Web of Science databases. We considered the period from 2000 to 2022, and we used the following word combinations: “cognitive evaluation” AND “severe brain injury” OR “severe traumatic brain injury” OR “neuropsychological evaluation in traumatic brain injury” OR “traumatic brain injury” OR “neurocognitive evaluation” OR “assessment scales”. To obtain a complete search, we also analysed the references of the selected articles. Two reviewers (WT and MT) screened 300 studies, according to title, abstracts, and text; among these, 166 papers were initially selected and eventually 146 were included as they met the inclusion criteria (Figure 1).

Inclusion criteria were (i) adult patients (age ≥ 18), (ii) patients with severe TBI, (iii) other severe brain damages that may cause a state of coma for at least 24 h, and (iv) a GCS score between 3 and 8. The exclusion criteria were (i) individuals with mild to moderate stroke and TBI, (ii) patients with previous psychiatric disorders (depression, anxiety, psychosis, delirium, and (iii) previous neuropsychological impairments.

## 3. Results

Our review included articles dealing with the neurocognitive rating scales most used by clinicians and researchers, according to the cognitive domain to investigate and the level of disability (Table 1, Table 2 and Table 3). In fact, it is necessary to distinguish between scales administered in acute/postacute and chronic phases as well as in patients with moderate-to-severe disability.

We divided our results into two main sections. The first, Section 3.1, deals with the screening tools used to test global cognition in the acute (like CSR-R or LCF) and postacute phase (like Mini Mental State Examination—MMSE and Montrel Cognitive Assessment—MoCA). In this section we reported five studies [35,36,37,38,39]. The second, Section 3.2, reports the neuropsychological assessment investigating specific cognitive domains. Notably, in this section we found (i) four articles related to the assessment of memory functions [19,20,21,22]; (ii) two articles assessing attention functions [23,24]; (iii) five articles that evaluated executive functions [26,27,28,29]; (iv) three articles dealing with social cognition [40,41,42]; (v) three studies about the evaluation of language disorders [30,31,32]; and (vi) only one study [43] assessing limb apraxia.

### 3.1. Global Cognitive Assessment

During the acute period after sTBI, there is often a great deal of uncertainty regarding the extent of cognitive and physical recovery as well as the long-term functional outcome. In this condition, only a few observational scales can be administered, and these are based on the evaluation of the patient’s spontaneous behaviours (eye opening, reflex responses, visual pursuit) or response to simple commands. For instance, by using the CRS-R, the examiner obtains a score that allows them to classify the patient within two main categories: VS/UWS or MCS (plus or minus). Conversely, using the LCF, it is possible to frame the patients in one of eight levels, from unconsciousness to normal consciousness. During the acute period, the most reliable prognostic clinical indicators are vegetative state duration, the duration of post-traumatic amnesia, and the CRS-R score [44,45,46]. The score of CRS-R reaches a sensitivity of 93% and a specificity of 96%. In fact, the CSR-R allows patients with DOC to be accurately differentiated from those that do not manifest alterations in conscious awareness. Additionally, clinicians should adopt a cut-off score of 8 to perform an appropriate differential diagnosis, since it offers the best odds of concurrently avoiding false-positive and -negative errors. However, the most accurate diagnosis is performed when the full subscale profile is available [35].

The Galveston Orientation and Amnesia Test (GOAT) investigates the presence of post-traumatic amnesia (PTA) following an sTBI. Some studies have identified the GOAT scores as a good predictor of long-term outcomes [36,37]. Specifically, when sTBI patients recover from an agitation state, clinicians should consider the use of screening tools to investigate cognitive functions globally with the administration of the MMSE and the MoCA, which are the most widely used means to this end.

Generally, these global assessment tests allow complete and fast administration, maximizing patients’ cooperation and reducing fatigue effects. Tay et al. [38] showed that MoCA has good reliability in both the inpatient and outpatient settings after TBI. It can be considered a valid tool to determine cognitive impairments in postacute traumatic events. It seems that MoCA presents both a sensitivity and specificity, respectively, of 79.4% and 74.1%. However, it still lacks sensitivity for estimating mild cognitive impairments. The Repeatable Battery for the Assessment of Neuropsychological Status (RBANS) has proven a very useful tool for screening a variety of cognitive domains in a relatively brief amount of time, and the current results suggest that the RBANS has a good sensitivity and specificity in assessing the neurobehavioural sequelae in TBI patients [39].

### 3.2. Specific Cognitive Domains in Neuropsychological Assessment

The different cognitive domains may be investigated using a wide range of psychodiagnostics tools that the neuropsychologist can choose based on the clinical picture and the patient’s needs [47]. Neuropsychological evaluation in TBI patients is of extreme importance, since, in addition to motor symptoms, cognitive deficits are also very disabling [48]. Most of the instruments listed below require the active participation of the subject, so they are suggested in those TBI patients who have recovered consciousness.

#### 3.2.1. Evaluation of Memory Deficits

TBI patients present a wide range of memory impairments, including working, prospective, semantic, and episodic as well as the encoding, storage, and retrieval of words. For these reasons, it is useful to consider an assessment battery that includes all subtypes of memory or to choose the appropriate tests for the domain that is most severely impaired in the patient.

In detail, Ashendorf et al. [19] administered the Rey–Osterrieth Auditory Verbal Learning Test (RAVLT), revealing its usefulness in assessing learning memory, by means of consolidation of new verbal information. Otherwise, the Rey–Osterrieth Complex Figure Test (RCFT) provides an assessment of visuospatial memory, associated with praxis skills. Carlozzi et al. [20] performed a memory evaluation in subjects with TBI (mild, moderate, and severe) using the Wechsler Memory Scale (WMS-IV), showing great capacities in distinguishing sTBI subjects from controls. On the other side, other authors [21,22] used the Digit Span to assess memory skills in TBI patients, finding a strong correlation between reduced working memory function and executive dysfunction (see Table 1).

#### 3.2.2. Assessment of Attention Deficits

Attention is the behavioural and cognitive process of selectively concentrating on a discrete stimulus while ignoring other perceivable stimuli [49]. Attention components include divided (carrying out several tasks at the same time), selective (ability to focus on a single stimulus in the presence of distractors), and sustained (ability to remain focused on a stimulus for a long period of time), which are often associated with mental slowness [50]. Attention deficits (ADs) are one of the most common cognitive impairments after a brain injury. The prevalence of ADs in the acute phase after sTBI ranges between 45% and 88%, while in the postacute phase, i.e., at discharge from hospital, the prevalence of these complaints is between 25% and 51% [49]. Patients with ADs often present with diminished concentration, distractibility, reduced error control, mental slowness, and fatigability. In addition, an AD is often associated with memory and language impairments [50]. Jourdan et al. [23] found that ADs were reported by patients at a 4 year-follow-up after brain injury and included mental slowness and concentration difficulties (56.7% of the patients) as well as dual-task difficulties (51.7%). Different tests may be used in the assessment of attentional functions in sTBI patients (see Table 2).

Neuropsychological tools that can detect ADs in this patient population include the continuous performance test (CPT), which measures different aspects of attention, although focusing on selective attention processes, and the Symbol Digit Modalities Test (SDMT), an instrument evaluating attention, perceptual speed, motor speed, and visual scanning [23,24]. To differentiate the components of attention, [51] the Paced Auditory Serial Addition Task (PASAT) can also be used, which is a neuropsychological test measuring auditory information, processing speed and flexibility, as well as calculation ability. Indeed, mental slowness is significantly correlated with injury severity and task complexity. Although it is a common opinion that mental slowness affects test scores, this is not particularly true in TBI patients, as shown by some studies [52]. According to the literature, sustained, divided, and selective attention in sTBI patients could be assessed with the popular Stroop test, as confirmed by some authors [53]. In fact, Stroop interference was significantly greater in TBI groups and scores were influenced by mental slowness and changes in sensory-speed processing. Ben-David et al. [53] reported that the presence of specific impairments in attentional functioning, such as divided attention, could depend on the complexity of the task.

#### 3.2.3. Evaluation of Executive Dysfunctions

The frontal lobes are known to play a key role in cognition, with regards to executive functions, i.e., the ability to engage in intentional, planned, and goal-directed behaviour. Executive dysfunction (ED) following sTBI is a well-documented problem, and it is often the most disabling aspect [54]. Some authors [55] compared the sensitivity of conventional tests for executive functions with a new one, called the Behavioral Dysexecutive Syndrome Inventory (BDSI). They found that 87% of patients showed ED, but 49% also presented a combination of behavioural and cognitive impairments. sTBI patients with ED also showed greater difficulties in community reintegration post injury, because of their decreased socialization, vocational loss, and increased family burden [56]. In particular, patients following sTBI frequently lost the ability to sort items into a specific category as demonstrated using the Wisconsin Card Sorting Test (WCST) [25,26,27].

Another tool frequently used in these patients is the Tower of London, which targets planning abilities [28]. During the assessment, clinicians should also consider “inhibition” (that is, the ability to control interference stimulus), which could be evaluated with the Stroop test [57]. Other authors investigated the role of using the Dysexecutive Questionnaire (DEX) in sTBI patients, finding that the DEX score was strongly correlated with cognitive alterations, mood disorders, and the ability to carry out simple activities of daily living [29] (see Table 2).

#### 3.2.4. Social Cognition and TBI Assessment

Social cognition refers to the ability to figure out people’s behaviour and to respond appropriately according to social situations [58,59]. Some authors found that sTBI patients tend to manifest alterations in recognizing facial expressions and emotions. However, the relationship between social cognition and neurocognitive deficits is still under debate [60].

The most used tests for the evaluation of social cognition in TBI patients are The Awareness of Social Inference Test (TASIT) [40] and The Social Decision-Making Task (SDMT) [41]. The TASIT is an audio–visual tool designed for the clinical assessment of social perception with alternate forms for retesting, and it is used to evaluate the ability of patients to understand emotional states, thoughts, intentions, and conversational meaning in everyday exchanges [42]. Notably, the TASIT in sTBI should be administered with caution because this patient population shows deficits in working memory, learning new tasks, and ED [40]. In this context, memory tasks related to social information, like remembering faces or stories, were considered more related to social perception. Otherwise, the SDMT offers a new way of examining decision making within a social context, especially following TBI. Kelly et al. [41] assessed patients affected by sTBI, comparing them with healthy controls using the Iowa Gambling Task (IGT) and a battery of neuropsychological tests and social cognition tasks. To summarize, the SDMT was found to be more sensitive in distinguishing people with TBI and healthy controls. However, performances on SDMT and IGT are not associated since they measure different constructs [61].

#### 3.2.5. Language Impairments and Assessment

Commonly, the most used assessment tools for language impairment in TBI patients include the Western Aphasia Battery-Revised (WAB-R) [30], the Aachener Aphasia Test (AAT) [62], the Boston Diagnostic Aphasia Examination (BDAE) [32], and the Boston Naming Test (BNT) [31]. In specific situations, clinicians can administer the BDAE test, which is considered the most representative to tailor assessment process and reduce testing time (especially in patients with TBI that often have AD), while preserving diagnostic sensitivity [32]. Rather than aphasia, about 80% of individuals with TBI have disorders in the interactional use of language and at discourse level [62]. These impairments are subsumed under the term “Cognitive Communication Disorders (CCD) [63] that demonstrates how “discourses are more than just a string of sentences”, with verbal macrostructures that are realized as either monologues or interactive dialogues. Research on discourse production in TBI has shown that working memory, attention, and ED are crucial for the construction of coherent verbal macrostructures [64,65].

A proper assessment of post-sTBI language disorders should therefore not exclude an analysis of discourse and narrative skills, and should evaluate macro-components (quantity of concepts expressed) and micro-components (total number of words, density, words per minute) [66]. A tool that provides an accurate investigation of this aspect is the “Protocole Montreal d’évaluation de la communication” (MEC) consisting of a 10 min conversation on two different topics with the examiner that is then rated on a 17-point checklist [67]. However, it is important for a clinician to carefully choose the tool to be adopted according to the severity and evolution of the TBI. It is also important to evaluate the long-term outcomes since communication disorders also have a negative impact on social relationships [63]. The most used tools to investigate language functions in TBI patients are shown in Table 3.

#### 3.2.6. Visuospatial and Praxis Assessment

People with TBI can present limb apraxia, which is a disorder in performing purposeful skill movements. The evaluation of these specific apraxia components has not been fully investigated, since the literature about the psychometric issues in testing limb apraxia is lacking. However, some authors identified some scales that can be useful in assessing apraxia in TBI patients, like the Western Aphasia Battery, the Boston Diagnostic Aphasia Exam, the Apraxia Battery for Adults-II (ABA), and the Florida Apraxia Screening Test-Revised (FAST-R) of the Florida Apraxia Battery (FAB) [43]. It should be considered that sTBI patients with limb apraxia are more likely to show concomitant cognitive dysfunctions in multiple domains, such as language, planning, imagery, inhibition of distraction, declarative memory, working memory, initiation, and overcoming fatigue [43]. This issue should be considered when dealing with apraxia assessment in sTBI.

## 4. Discussion

Neuropsychological assessment is a useful process able to detect and monitor global neuropsychological deficits or impairments in specific cognitive domains, especially in sTBI patients where cognitive dysfunctions are present in almost all affected subjects [68]. The novelty of this review consists of having comprehensively introduced a wide set of test batteries that evaluate and fully explore the patient’s functioning from the state of coma to the possible functional recovery following sTBI. In fact, the severity of the injury is decisive for the development of cognitive sequelae, as confirmed by Tsai et al. [69], who highlighted a prevalence of 40 to 60% of memory and attention deficits, in which 1–3 still had memory impairments years after the brain injury, and they may depend on coma length. In addition, it was found [70,71] that TBI patients are more likely to develop verbal or visual memory tasks, especially in the retrieval of both semantic and autobiographical information. As regards working memory, it has been demonstrated that task accuracy is poorer in subjects with TBI [72]. According to Dunning et al. [73], patients affected by TBI manifested significant deficits in verbal short-term memory and visuospatial and verbal working memory. On the other hand, Mioni et al. [74] assessed memory functions in sTBI patients, showing that their performances were related to executive functions. Several studies support the idea that cognitive impairment affects long-term functional status after TBI [33]. Notably, adequate assessment in TBI patients allows the implementation of their care path and establishes specific therapeutic and rehabilitative programs [16]. Indeed, sTBI patients may recover gross motor functions although they still maintain neuropsychological/behavioural problems months or years after the brain injury, affecting social and family relationships. Lesions of the frontal lobes seem to cause the most severe form of cognitive deficit, as these involve executive function. Executive dysfunction represents the most disabling cognitive deficit in patients with TBI, since it affects self-regulation, planning skills, and affective lability. In this context, clinicians should consider the differential diagnosis between ED and limb apraxia. It is not uncommon that motor and executive components often overlap with spatial aspects in construction tasks [34]. In fact, visuospatial assessment usually does not involve directly executive components, such as in the ROFC, in which the patient must copy a complex project and evaluate the respondent’s ability to use signals to retrieve information [19]. Benton’s spatial orientation test (BSOT) does not have a strong executive control component, like the Benton visual retention test (BVRT) in which the patient is briefly shown cards containing two or three geometric designs and then asked to reproduce them by heart. This test evaluates visual perception, short-term visual memory, and visuo-constructive ability [75]. On the other hand, other tests, i.e., the clock drawing test (CDT) [76] and the judgement of line guidance (JOL) [77], have a strong visuospatial and executive control component. Although these tests have been tailored to patients with neurodegenerative diseases, they could probably also be used in TBI patients.

What is more, language disorders and aphasia evaluation should be addressed by clinicians, since the incidence is quite frequent, ranging from 2% to 32% [78,79], and involves difficulties in naming and word-finding, sentence comprehension, or sentence production [79]. For this reason, in many cases, the use of aphasia batteries, which evaluate vocabulary and grammatical abilities at the single-word and sentence levels, may not be sensitive to the evaluation of these impairments [30,32,62].

Lastly, the importance of the method of test administration should not be underestimated. Face-to-face interaction within the paper–pencil modality is the most used. However, the psychometric properties of these conventional neuropsychological tests, especially those to investigate ED, have been widely discussed. In fact, TBI patients may have good scores in executive tests, although they manifest serious ED in daily life activities [55,80]. This is why some researchers have suggested using tasks with an open-ended structure, such as going shopping, finding one’s way in natural surroundings, preparing a meal, and dealing with a complex multitasking situation [81]. To overcome this concern, clinicians can consider using a PC-based/telecognitive virtual assessment for providing healthcare services at distance, which remotely connect patients and health professionals. Currently, these advanced assessment and treatment tools have proven essential during the pandemic to ensure continuity of care, increasing motivation and the patient’s positive mood [82,83].

Several tests and batteries are available to date, and it is important to understand how they are used and can be interpreted appropriately to properly interpret the results to maximize patient outcomes. In this vein, the International Mission for Prognosis and Analysis of Clinical Trials in TBI study group and the development of uniform data standards named common data elements have supported a broad list of tests and batteries. Notably, this protocol can be administered in TBI patients, divided into sub-categories: “concussion/mild TBI”, “acute hospitalized”, and “moderate/severe TBI: Rehabilitation” [83,84]. To this end, TBI is one of the most heterogeneous neurological disorders which makes the assessment standardization process really challenging, but also points out the need for a better definition of clinical assessment of TBI subgroup severity level.

The main limitation of this scoping review that needs to be addressed is the absence of quality and risk-of-bias assessments in the selected papers. However, our aim was to investigate the available literature about the neuropsychological tests/scales used to evaluate sTBI patients, also providing some clinical advice. In the near future, a systematic review should be considered to better point out the validity of studies and the reliability of the scales, since the literature is still lacking on this issue. Moreover, a quality assessment of the studies should be performed (e.g., by using the pyramid of evidence) to provide the reader with clearer information on the quality of the manuscripts selected.

## 5. Author’s Clinical Advice

Herein we present some clinical advice/suggestions for performing neuropsychological assessment, for both moderate and sTBI, according to the updated literature. In fact, the clinical neuropsychological assessment in this patient population is poorly standardized, especially concerning the distinction between moderate and severe TBI.

During the first phase following coma (acute phase 0–1 month), responsiveness monitoring can be carried out using some scales. In detail, the CRS-R is considered as the gold-standard in this phase for its psychometric parameters. The reliability of the scale is improved when there is the presence of caregiver during the assessment, as this represents an important emotional stimulus for patients [85].

After the acute phase, the difference between moderate and severe TBI is more pronounced. In fact, patients with moderate TBI tend to show improvements in the first months after the acute phase. In the postacute (1–3 months) phase, when and if consciousness and cognitive activity improve, clinicians should monitor the patients’ cognitive status through observation of behaviours, vigilance, and/or spontaneous speech [86]. After that, the possible administration of a general examination through MMSE [87,88] and/or MoCA [89] can be considered to evaluate the patient’s global neurocognitive and functional status. Additionally, the assessment of visuospatial praxis can be addressed through CDT [76]; however, the moderate and/or severe TBI patient must not have aphasia or severe ideomotor praxis.

In the chronic phase (6 months), both moderate and severe TBI patients could be tested only when they regain sufficient cognitive status. It should be considered that the in-depth cognitive evaluation is based on results achieved for MMSE and/or MOCA; otherwise, the domain-specific diagnostic process might be incoherent [87,88]. In clinical practice, the administration of RAVLT [19], CPT [23], SDMT [24], PASAT [51], and Stroop [18] to evaluate frontal abilities can be very challenging, due to the lack of compliance of patients and the presence of language disturbances. This is why those tests should be administered in TBI patients with a moderate cognitive impairment level. Similarly, for the assessment of praxis skills, some clinicians use the FAST-R. However, the drawing and copying tasks can be easily administered to assess constructive apraxia [90], while the gesture-imitation test is used to evaluate ideomotor apraxia [43]. In cases in which the patient presents the optimal condition (active participation), it is possible to proceed using second-level batteries to investigate the “deeper cognitive functions” for specific domains. It is noteworthy that first-level test batteries, administered in sTBI, can be divided into two or more evaluation sessions; alternatively, it is possible to choose the tests to be performed based on the specific cognitive domain that is going to be explored. It is advisable to use shorter clinical evaluation batteries to avoid the fatigue, distractibility, and flooring effects typical of these patients.

In the end, clinicians should consider some tips in order to perform the most valid neuropsychological assessment in sTBI. In detail, the testing needs to be administered in a lab or clinic office setting, with minimal noise and distraction (because of the high prevalence of attention deficits in TBI), where all testing is performed one-on-one. As many specific neuropsychological tests are available, they should be administered only in TBI patients who have recovered sufficiently to cooperate with testing [91]. In fact, DOC as well as potentially severe motor [92] and language impairments make some sTBI individuals inappropriate for neuropsychological referral because of a lack of testability. In this vein, clinicians should be aware of the demotivating effect and the patient’s effort to avoid test invalidity. This is why neuropsychological tests can be marked up as “invalid” in two cases: (1) individuals not motivated to perform and thus not “trying hard” and (2) individuals attempting to exaggerate impairment by answering incorrectly or feigning deficits. Poor effort can be caused by factors other than TBI.

Lastly, clinicians should monitor and evaluate patients after eight months or one-year post injury, since this is the period in which natural brain recovery can still occur. In this vein, sustained and persistent symptoms in TBI patients should be screened for anxiety and depression, as they are the most powerful predictors of prolonged recovery [93,94]. Specifically, brief standardised self-report measures such as the Generalised Anxiety Disorder and Patient Health Questionnaire can facilitate screening [93,94].

## 6. Conclusions

In summary, neuropsychological evaluation in TBI provides a comprehensive assessment of patients’ cognitive strengths and weaknesses, and this should be administered by using different tools at the different stages of the disorder. However, since all cognitive tests require that patients give their best effort when completing them and patients with sTBI present a complex clinical picture, more methodologically rigorous research is needed to demonstrate the sensitivity/specificity of the tools to identify which patients are most likely to respond. A systematic review with a metanalysis overcoming this important consideration is needed to better point out the validity of the papers, and the reliability of the scales, in order to guarantee a global holistic approach to these very frail individuals.

## Figures and Tables

**Table 1:** Memory assessment in TBI patients.

Reference n°	Sample Size	Assessment Description	Major Findings
Learning memory			
[19]	37 patients with severe TBI	The Rey Auditory Verbal Learning Test (RAVLT) is used to measure delayed recall and recognition memory, while the Rey–Osterrieth Complex Figure Test (ROCF) is a neuropsychological assessment tool commonly used to measure the visuo-constructional and visual memory abilities of neuropsychiatric disorders, including copying and recall tests. By drawing the complex figure, the dysfunctional decline of an individual in multiple cognitive dimensions can be evaluated, such as attention and concentration, fine-motor coordination, visuospatial perception, nonverbal memory, planning and organization, and spatial orientation.	ROCF Recognition Hits and MEP displayed at least acceptable discriminant strength with 35% sensitivity and at least 90% specificity.
Visual memory			
[20]	100 patients with TBI (*n* = 35 complicated mild or moderate TBI; *n* = 65 severe TBI)	The Wechsler Memory Scale (WMS-IV) is a neuropsychological test built to assess different memory functions. The last version is the fourth edition (WMS-IV) which was published in 2009 and which was designed to be used with the WAIS-IV. Performance is reported as five index scores: Auditory Memory, Visual Memory, Visual Working Memory, Immediate Memory, and Delayed Memory.	WMS- IV also showed good sensitivity and specificity for classifying individuals with severe TBI versus controls, but not for classifying individuals with memory impairments relative to those without (there was adequate specificity but poor sensitivity).
Short-term memory/working memory			
[21]	64 patients with moderate-to-severe TBI (TBI)	The Digit Span Forward/Backward (DSF/DSB) is a measure of verbal short-term and working memory that can be used in two formats, DSF and DSB. It is a verbal task, with stimuli presented auditorily, and responses spoken by the patients.	TBI patients are more likely to perseverate on prior instructions during DS sequencing.
[22]	30 patients with severe chronic TBI	Digit Span (see description above)	Severe TBI patients are associated with an impairment of executive aspects of working memory. The anatomic substrate of this impairment remains to be elucidated. It might be related to a defective activation of a distributed network, including the dorsolateral prefrontal cortex.

Legend: RAVLT (Rey Auditory Verbal Learning Test), ROCF (Rey–Osterrieth Complex Figure Test), WMS-IV (Wechsler Memory Scale), DSF (Digit Span Forward), DSB (Digit Span Backward).

**Table 2:** Assessment of attention and executive functions in TBI.

Reference n°	Sample Size	Assessment Description	Major Finding
Attention			
[23]	30 moderate-to-severe TBI patients	The continuous performance test (CPT) is a valuable way to measure different aspects of attention. During a CPT, the user is instructed to respond only when the target stimulus is presented and to withhold responses to other stimuli. Stimuli may be visual, auditory, or both simultaneously.	The CPT-II has validity for use as an attentional measure among patients with TBI.
[24]	Twenty-five patients with complicated mild-to-severe BI	The Symbol Digit Modalities Test (SDMT) is a screening tool usually used in clinical and research settings to evaluate neurological dysfunction. Like other substitution tasks, performance on the SDMT is underpinned by attention, perceptual speed, motor speed, and visual scanning.	The present data strongly support the pervasive influence of reduced speed of information processing on attentional performance after TBI.
Executive functions			
[25]	29 severe TBI patients (9 females and 20 males)	The Wisconsin Card Sorting Test (WCST) is a neuropsychological test that is usually used to assess such higher-level cognitive processes as attention, perseverance, working memory, abstract thinking, and set shifting. The Delis–Kaplan Executive Function System (D- KEFS) is a neuropsychological clinical battery used to assess high-level cognitive functions such as verbal and nonverbal executive domains in nine different areas.	Inter-rater reliability showed the translation to be reliable and effective. The D-KEFS ST can effectively distinguish TBI patients from control subjects, with the TBI group consistently demonstrating difficulties in category/concept formation and in flexibility of thought.
[26]	176 patients with TBI	WCST (see the description above)	Results suggested a dose–response relationship between TBI severity and deficits on the WCST in patients providing good effort during testing.
[27]	NA	WCST (see description above)	Although there are shorter and/or automated variations, the classic WCST with 128 cards is still the most popular. The WCST is a helpful tool for clinical and research applications; however, it is usual practice to report only one or a small number of potential values, preventing further accurate comparisons between studies.
[28]	56 patients with complicated mild-to-severe TBI	The Tower of London (TOL) task has been used largely as a test of planning ability in neuropsychological patients. Patients are asked to preplan mentally a sequence of moves to match a start set of discs to a goal, and then to execute the moves one by one.	Poor sensitivity of this measure limits its use in isolation; the TOLDX may provide a complementary measurement of aspects of problem-solving deficit in TBI that may not be captured by other tests.
[29]	Sixty-three patients (and relatives) were included within 63.4 months (±20.7) after sTBI	The dysexecutive questionnaire (DEX) was designed to assess different domains of executive functioning in daily life.	Executive function, episodic memory, attention (phasic alert sustained and divided attention), the GOSE, and the volume of the corpus callosum (an MRI marker) were all substantially linked with DEX-O. The mean diffusivity measurement was associated with the anosognosia score (DEX-O minus DEX-S). These findings demonstrate DEX-O’s clinical use in determining long-term impairment.

Legend: (CPT) continuous performance test, (SDMT) Symbol Digit Modalities Test, (WCST) Wisconsin Card Sorting Test, (D-KEFS) Delis–Kaplan Executive Function System, (TOL) Tower of London, (DEX) Dysexecutive questionnaire.

**Table 3:** Assessment of language/speech in TBI patients.

Reference n°	Sample Size	Assessment Description	Major Findings
Language–Speech			
[30]	169 TBI patients	The Western Aphasia Battery-Revised (WAB-R) measures linguistic skills most frequently affected by aphasia, plus key nonlinguistic skills, and provides differential diagnosis information. Flexible to various administration settings from hospital room to clinic, it provides a baseline level of performance to measure change over time.	WAB-R alone is insufficient to detect or fully characterize aphasia and the motor speech deficits that may accompany speech impairment; it should be considered for use only as one component of a larger communication assessment battery.
[31]	Twenty patients with moderate-to-severe TBI	The Neuropsychological Assessment Battery (NAB) is a comprehensive test battery that assesses five cognitive domains (Attention, Language, Memory, Spatial, and Executive Functions).	NAB demonstrated by the current sample is consistent with the neuropsychological profile observed in postacute patients with moderate-to-severe TBI without focal deficits (e.g., aphasia), demonstrating its relative sensitivity in this patient population.
[32]	355 TBI patients with aphasia	A well-known test battery that looks at several aspects of language functioning is the BDAE-3 version. The BDAE-3 is a more thorough aphasia battery as compared to other aphasia batteries since it includes more than 50 subtests and may be interpreted using the Boston Process Approach [33,34]. BDAE-3 offers strong validity and reliability for assessing patients with severe TBI.	Across different degrees of aphasia, the BDAE-3 shows high construct validity, and specific language functions (such as receptive vs. expressive language) continue to be important, separate language domains.

Legend: The Western Aphasia Battery-Revised (WAB-R), The Neuropsychological Assessment Battery (NAB), Boston Diagnostic Aphasia Examination (BDAE).

## Data Availability

Not applicable.

## Abbreviations

AAT	Aachener Aphasia Test
ABA	Apraxia Battery for Adults-II
AD	Attention Dysfunction
BDAE	Boston Diagnostic Aphasia Examination
BNT	Boston Naming Test
BSOT	Benton’s spatial orientation test
BVRT	Benton’s visual retention test
CCD	Cognitive Communication Disorders
CDT	Clock drawing test
CPT	Continuous performance test
CRS-R	Coma recovery scale-revised
DEX	Dysexecutive questionnaire
D-KEFS	Delis–Kaplan Executive Function System
DoC	Disorder of Consciousness
DRS	Disability Rating Scale
ED	Executive Dysfunction
FAB	Florida Apraxia Battery
FAB	Frontal Assessment Battery
FAST-R	Florida Apraxia Screening Test-Revised
GCS	Glasgow coma scale
GOAT	Galveston Orientation and Amnesia Test
IGT	Iowa Gambling Task
JOL	Judgement of line guidance
LCF	Level of Cognitive Functioning
MCS	Minimally Conscious State
MEC	Protocole Montreal d’évaluation de la communication
NAB	Neuropsychological Assessment Battery
PASAT	Paced Auditory Serial Addition Task
PTA	Post-traumatic amnesia
SDMT	Symbol Digit Modalities Test
SDMT	Social Decision-Making Task
TASIT	The Awareness of Social Inference Test
TBI	Traumatic brain injury
TOL	Tower of London
UWS	Unresponsive wakefulness syndrome
VS	Vegetative state
WAB-R	Western Aphasia Battery-Revised
WCST	Wisconsin Card Sorting Test
WMS-IV	Wechsler Memory Scale
